# Effects of a Berry Polyphenolic Fraction on the Pathogenic Properties of *Porphyromonas gingivalis*

**DOI:** 10.3389/froh.2022.923663

**Published:** 2022-06-16

**Authors:** Katy Vaillancourt, Amel Ben Lagha, Daniel Grenier

**Affiliations:** Oral Ecology Research Group, Faculty of Dentistry, Université Laval, Quebec City, QC, Canada

**Keywords:** *P. gingivalis*, periodontal disease, gingipain, polyphenol, keratinocyte

## Abstract

*Porphyromonas gingivalis* expresses a broad array of virulence factors that enable it to play a central role in the etiopathogenesis of periodontitis. The objective of the present study was to assess the effects of a berry polyphenolic fraction (Orophenol®) composed of extracts from cranberry, wild blueberry, and strawberry on the main pathogenic determinants of *P. gingivalis*. Orophenol® attenuated the growth of *P. gingivalis* and decreased its hemolytic activity, its adherence to a basement membrane matrix model, and its proteinase activities. The berry polyphenolic fraction also impaired the production of reactive oxygen species (ROS) by oral keratinocytes stimulated with *P. gingivalis*. Lastly, using an *in vitro* model of oral keratinocyte barrier, the fraction exerted a protective effect against the damages mediated by *P. gingivalis*. In conclusion, the berry polyphenolic fraction investigated in the present study attenuated several pathogenic properties of *P. gingivalis*. Although future clinical investigations are required, our study provided evidence that the polyphenols contained in this fraction may represent bioactive molecules of high interest for the prevention and/or treatment of periodontal disease.

## Introduction

The Gram-negative strictly anaerobic bacterium *Porphyromonas gingivalis* is a member of the human oral microbiome and can be regarded as an opportunistic pathogen [1]. *P. gingivalis* can promote a state of dysbiosis resulting in an imbalance between beneficial commensal bacteria and periodontopathogenic bacteria [2]. It is often isolated concomitantly with *Tannerella forsythia* and *Treponema denticola*, forming the red complex initially described by Ximenez-Fyvie et al. [3], and is strongly linked with the chronic form of periodontitis. This bacteria-induced inflammatory disease, which affects tooth-supporting tissues, is characterized by the formation of periodontal pocket, along with loss of clinical attachment and resorption of the alveolar bone. Over the past decade, studies have established a link between *P. gingivalis* and several systemic disorders, including atherosclerosis, Alzheimer's disease, and rheumatoid arthritis [4, 5]. The ability of this periodontal pathogen to invade the ulcerated oral epithelium and translocate from the oral cavity to distant tissues or organs might explain *P. gingivalis*-associated systemic disorders [4, 5].

*P. gingivalis* expresses a broad array of potential virulence factors that are involved in subgingival colonization, immune defense evasion, connective tissue destruction, and exaggerated inflammatory responses [6, 7]. The pathogenic factors produced by *P. gingivalis* include lipopolysaccharides (LPS), adhesins, hemolysins, and proteinases [6, 7]. Gingipains, which are either secreted or anchored to the outer membrane, are the main proteinases produced by *P. gingivalis* [8, 9]. Targeting virulence factors is a promising strategy for specifically fighting bacterial pathogens without disturbing the commensal microbiota that often has a beneficial impact [10, 11].

Although a satisfactory therapeutic outcome is commonly achieved with a conventional mechanical treatment, controlling periodontitis in high-risk subjects (smokers, diabetics, elderly populations, etc.) or patients with an aggressive form of the disease often needs an adjunctive treatment with systemic or locally applied antibiotics that may lead to the emergence of bacterial resistance [12, 13]. In recent decades, research has been directed at discovering natural compounds that display beneficial properties with respect to periodontal health as efficient alternatives to conventional antibiotics [14–16]. Polyphenols refer to different families of compounds in edible plants such as fruits and vegetables and with which diverse human health benefits have been linked [17]. These plant secondary metabolites are well-known to possess antibacterial, anti-adhesion, and anti-inflammatory activities that may be relevant with respect to periodontal health [14–18]. Wild blueberries (*Vaccinium angustifolium*), cranberries (*Vaccinium macrocarpon*), and strawberries (*Fragaria virginiana*) contain high amounts of phenolic acids, anthocyanins, and proanthocyanidins [19]. Previous reports have brought clear evidence that proanthocyanidins from berry fruits exhibit anti-biofilm and anti-adhesion properties against important oral pathogens [16, 20, 21]. The objective of the present study was to assess a berry polyphenolic preparation (Orophenol®) composed of extracts from cranberry, wild blueberry, and strawberry for its effect on the pathogenic properties of the periodontal pathogen *P. gingivalis*.

## Materials and Methods

### Berry Polyphenolic Fraction (Orophenol®)

The berry polyphenolic fraction, commercialized as Orophenol®, was kindly provided by the manufacturer Diana Food Canada Inc. (Champlain, QC, Canada). This fraction contains polyphenol-rich soluble extracts prepared from three berry fruits (cranberry, wild blueberry, strawberry). A stock solution was prepared in distilled water at a concentration of 10 mg/ml, was filter-sterilized (0.2-μm pore size membrane filter), and was kept at 4°C under darkness for up to 1 month. The phenolic content of the preparation was analyzed by chromatographic and mass spectrometry techniques and has been detailed in a previous study [22]. In brief, phenolic acids, flavonoids (flavonols, anthocyanins, flavan-3-ols), and procyanidins made up 10.71, 19.76, and 5.29% (w/w) of the berry polyphenolic preparation, respectively.

### Bacteria and Growth Conditions

*Porphyromonas gingivalis* ATCC 33277 was grown in Todd-Hewitt broth (THB; Becton Dickinson and Company, Sparks, MD, United States) containing 0.001% (w/v) hemin and 0.0001% (w/v) vitamin K and was incubated at 37°C in an anaerobic atmosphere (80% N_2_, 10% CO_2_, and 10% H_2_).

### Growth Assay

The effect of the berry polyphenolic fraction on the growth of *P. gingivalis* was assessed with a broth microdilution method. Briefly, a 24-h culture of *P. gingivalis* was diluted in fresh medium to an optical density at 660 nm (OD_660_) of 0.1, which corresponds to a concentration of 1 × 10^8^ colony forming units (CFU)/mL. Equal volumes (100 μl) of the bacterial culture and two-fold serial dilutions of the berry polyphenolic fraction (2,000–7.81 μg/ml) in fresh medium were mixed in the wells of a 96-well tissue culture treated, flat-bottom, microplate (Sarstedt, Newton, NC, United States). Wells without bacteria or fraction were used as controls. The microplate was incubated for 24 h under anaerobic conditions prior to assessing bacterial growth by recording the OD_660_ using a Synergy 2 microplate reader (BioTek Instruments, Winooski, VT, United States).

### Hemolytic Activity

Sheep blood (Nutri-Bact, Terrebonne, QC, Canada) was centrifuged (600 × *g* for 5 min) to harvest erythrocytes, which were washed three times in 50 mM phosphate-buffered saline (pH 7.2, PBS) and suspended [2% (v/v)] in PBS. Equal volumes (1 ml) of the red blood cell suspension, the *P. gingivalis* suspension (OD_660_ = 2.0 in PBS; corresponding to to a concentration of 2 × 10^9^ CFU/ml), and the berry polyphenolic fraction (final concentrations of 0, 1.95, 3.9, 7.81, 15.625, and 31.25 μg/ml) were mixed and were incubated at 37°C for 16 h and then at 4°C for 1 h. The assay mixtures were centrifuged (10,000 × *g* for 5 min), and the absorbance of the supernatants was monitored at 540 nm (A_540_). Hemolysis induced by *P. gingivalis* in the absence of the berry polyphenolic fraction was given a value of 100%.

### Adherence to a Basement Membrane Matrix Model

The effect of the berry polyphenolic fraction on the ability of *P. gingivalis* to adhere to Matrigel® (BD Biosciences, Franklin Lakes, NJ, United States), an *in vitro* basement membrane matrix model, was investigated. First, bacterial cells were labeled with fluorescein isothiocyanate (FITC) as previously described [23]. *P. gingivalis* was incubated (2 h) with the Matrigel®, contained into wells of a 96-well microplate, in the presence or absence of the berry polyphenolic fraction (1,000 to 15.625 μg/ml). The wells were then washed three times with PBS and bound bacterial cells were estimated by recording the relative fluorescence units (RUF; excitation wavelength 495 nm; emission wavelength 525 nm) using a Synergy 2 microplate reader (BioTek Instruments). A 100% value was assigned to bacterial adherence determined in the absence of the berry polyphenolic fraction.

### Collagenase Activity

The effect of the berry polyphenolic fraction on the *P. gingivalis* collagenase activity was investigated by mixing a 48-h bacterial culture supernatant with fluorescent collagen DQ (Molecular Probes, Eugene, OR, United States) (100 μg/ml) in the presence of the fraction (final concentrations in the assay: 31.25, 62.5, 125, 250 μg/ml) in the wells of a microplate with a clear bottom and black walls (Greiner Bio-One North America, Monroe, NC, United States). After a 60-min incubation at 37°C, the fluorescence (Ex: 495 nm/Em: 535 nm) resulting from collagen degradation was monitored using a Synergy 2 microplate reader (BioTek Instruments). The berry polyphenolic fraction or the fluorescent substrate alone served as controls. Leupeptin (100 μg/ml) was included as a known inhibitor of *P. gingivalis* collagenase. Collagenase activity monitored in the absence of the fraction was given a value of 100%.

### Gingipain Activity

To investigate the effect of the berry polyphenolic fraction on the activities of *P. gingivalis* Arg- and Lys-gingipains, a bacterial cell suspension (OD_660_ of 0.4 in PBS; corresponding to to a concentration of 4 × 10^8^ CFU/ml) was incubated at 37°C in the wells of a 96-well microplate together with 10 mM dithiothreitol and 5 mM of either N-α-benzoyl-DL-arginine-*p*-nitroanilide (Arg-gingipain substrate) or N-*p*-tosyl-glycine-proline-lysine-*p*-nitroanilide (Lys-gingipain substrate) and in the presence or absence of the fraction (final concentrations in the assay: 1.95, 3.91, 7.81, 15.625, 31.25, 62.5, 125, 250 μg/ml). The incubation (37°C) was carried out for 60 min (Arg-gingipain) or 18 h (Lys-gingipain). Cleavage of the gingipain substrates was quantified by monitoring the absorbance at 405 nm (A_405_) of the cell-free supernatants. Tosyl-L-lysine chloromethyl ketone hydrochloride (TLCK; 4 mM) was included as a positive gingipain inhibitor. Gingipain activity determined in the absence of the berry polyphenolic fraction was assigned a value of 100%.

### Matrix Metalloproteinase 9 Activity

The effect of the berry polyphenolic fraction (31.25, 62.5, 125, 250 μg/ml) on the catalytic activity of matrix metalloproteinase (MMP)-9 was investigated using a commercial MMP-9 Inhibitor Screening Fluorometric Assay Kit (Abcam Inc., Toronto, ON, Canada). MMP-9 activity was determined after a 20-min incubation at 37°C by monitoring fluorescence (Ex: 328 nm/Em: 420 nm) using a Synergy 2 microplate reader (BioTek Instruments). N-isobutyl-N-(4-methoxyphenylsulfonyl) glycyl hydroxamic acid (NNGH; 1 mM) served as a positive inhibitor control of MMP-9. A 100% value was assigned to the activity obtained in the absence of the berry polyphenolic fraction.

### Reactive Oxygen Species Production by Oral Keratinocytes

The human oral keratinocyte cell line B11 [24] was generously provided by S. Groeger and J. Meyle (Justus-Lieig-University Giessen, Germany). The cells were cultivated in keratinocyte serum-free medium (K-SFM; Life Technologies Inc., Burlington, ON, Canada) enriched with bovine pituitary extract (50 μg/ml), recombinant epidermal growth factor (5 ng/ml), and penicillin G-streptomycin (100 μg/ml). The ability of the berry polyphenolic fraction to impair the capacity of *P. gingivalis* to induce reactive oxygen species (ROS) production in oral keratinocytes was investigated using a fluorometric method that monitors the oxidation of 2', 7'-dichlorofluorescein-diacetate (DCF-DA (40 mM in DMSO); Sigma-Aldrich Canada Co., Oakville, ON, Canada) into a fluorescent compound. B11 oral keratinocytes were seeded in the wells (10^5^ cells/well) of a 96-well microplate with black walls and a clear flat bottom (Greiner Bio-One North America) and were incubated overnight at 37°C in a 5% CO_2_ atmosphere. The cells were washed with Hank's balanced salt solution (HBSS; HyClone Laboratories, Logan, UT, United States) prior to adding 100 μM DCF-DA in HBSS. After 30 min, the keratinocytes were washed twice with HBSS prior to treating with *P. gingivalis* (in HBSS) at a multiplicity of infection (MOI) of 10^3^ in the absence or presence of the berry polyphenolic fraction (500–7.81 μg/ml). The fluorescence emission at 528 nm, following excitation at 485 nm, corresponding to ROS production was monitored after a 6-h incubation at 37°C using a Synergy 2 microplate reader (BioTek Instruments). A 100% value was assigned to ROS production induced by *P. gingivalis* in the absence of the fraction.

### Transepithelial Electrical Resistance of Oral Keratinocytes

The protective effect of the berry polyphenolic fraction (500–7.81 μg/ml) against *P. gingivalis* (MOI of 10^3^)-mediated deleterious effects on oral keratinocyte barrier function was investigated using the B11 cell line according to the procedure described in a previous study [25]. The barrier integrity of the keratinocyte model was assessed by recording the transepithelial electrical resistance (TEER) after a 24-h incubation (37°C/5% CO_2_) using an ohmmeter (EVOM2, World Precision Instruments, Sarasota, FL, United States). A relative value of 100% was assigned to the TEER recorded in the absence of *P. gingivalis* and the fraction.

### Statistical Analysis

Unless indicated otherwise, all assays were carried out in triplicate in three independent experiments, and the means ± standard deviations (SD) were calculated. Data were analyzed for signifiance using a one-way ANOVA analysis of variance with a *post hoc* Bonferroni multiple comparison test (GraphPad Software Inc., San Diego, CA, United States). Results were considered statistically significant at *P* < 0.01.

## Results

**Figure 1:**
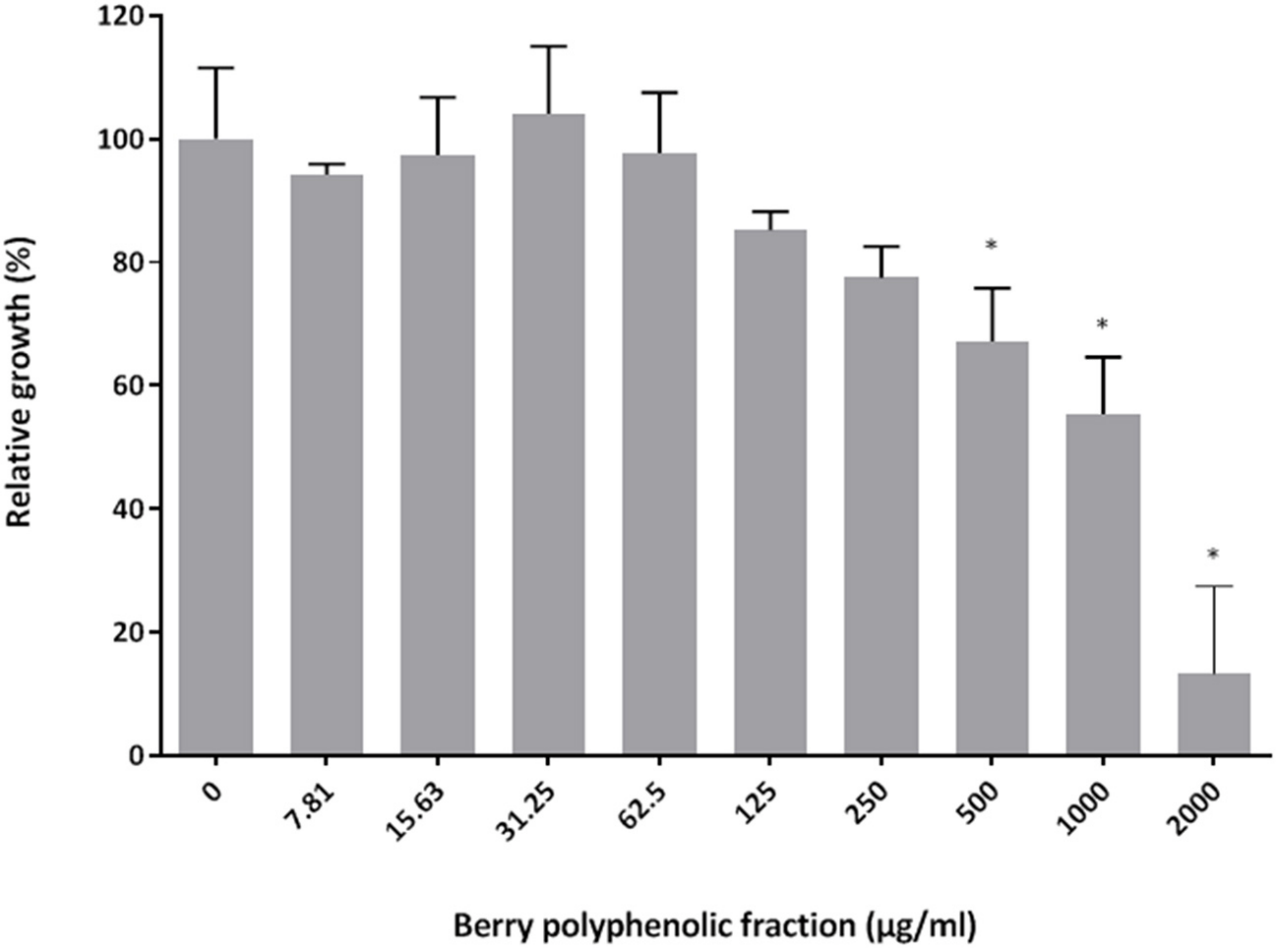
Effect of the berry polyphenolic fraction on the growth of *P. gingivalis*. A relative value of 100% was assigned to growth in the absence of the berry polyphenolic fraction. Results are expressed as the means ± SD of triplicate assays from three independent experiments. *Significant inhibition (*p* < 0.01) compared to the control.

The effect of the berry polyphenolic fraction on the growth of *P. gingivalis* ATCC 33277 was examined using a broth microdilution method. Figure 1 shows that the fraction reduced the growth of *P. gingivalis* in a dose-dependent manner. Significant inhibitions were obtained with concentrations of the berry polyphenolic fraction ≥500 μg/ml. At the highest concentration tested (2,000 μg/ml), the fraction lowered the bacterial growth by 86.7%.

**Figure 2:**
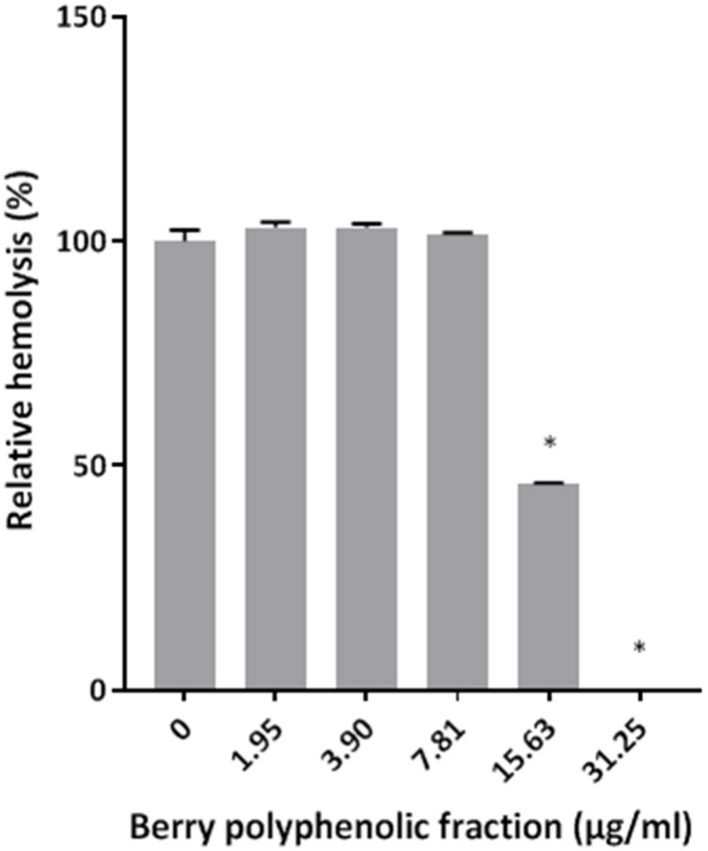
Effect of the berry polyphenolic fraction on the hemolysis of sheep red blood cells by *P. gingivalis*. A relative value of 100% was assigned to the hemolysis of sheep red blood cells in the absence of the berry polyphenolic fraction. Results are expressed as the means ± SD of triplicate assays from three independent experiments. *Significant inhibition (*p* < 0.01) compared to the control.

The effect of the berry polyphenolic fraction on the hemolytic activity of *P. gingivalis* was then investigated. As shown in Figure 2, the fraction dose-dependently inhibited hemolysis of sheep erythrocytes. Complete inhibition was obtained with the berry polyphenolic fraction at a concentration of 31.25 μg/ml.

**Figure 3:**
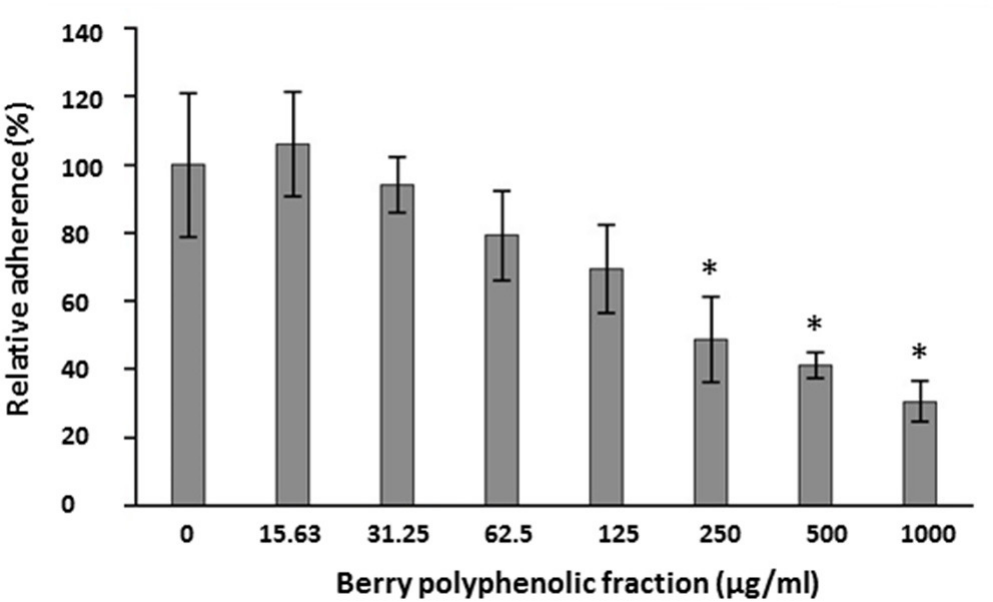
Effect of the berry polyphenolic fraction on the adhesion of *P. gingivalis* to the Matrigel® basement membrane model. A relative value of 100% was assigned to the adherence in the absence of the berry polyphenolic fraction. Results are expressed as the means ± SD of triplicate assays from three independent experiments. *Significant inhibition (*p* < 0.01) compared to the control.

The ability of the fraction to impair the adhesion of FITC-labeled *P. gingivalis* to a Matrigel® layer was examined. The fraction inhibited the adhesion of *P. gingivalis* to the basement membrane matrix model in a dose-dependent manner (Figure 3). The inhibition was significant at concentrations ≥250 μg/ml.

**Figure 4:**
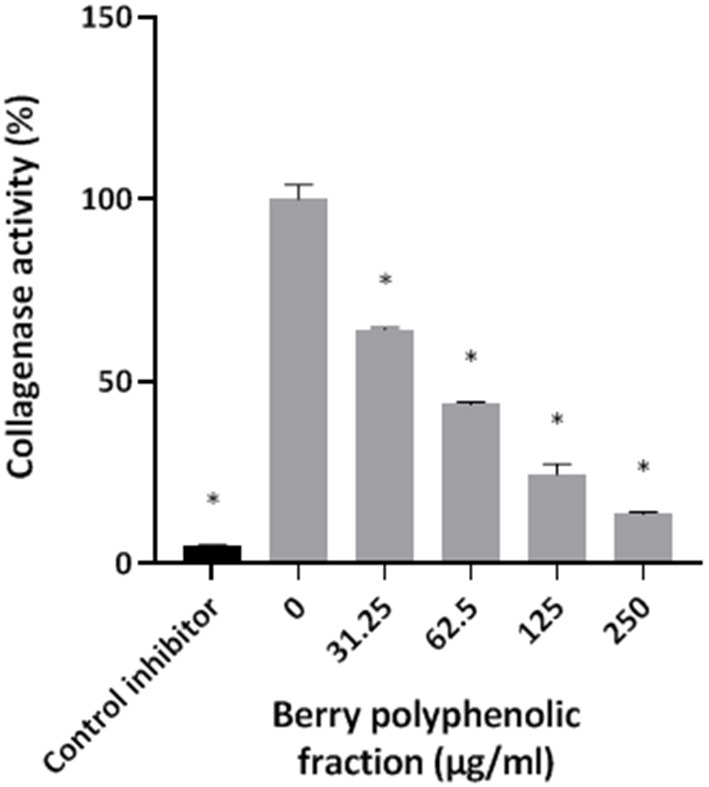
Effect of the berry polyphenolic fraction on the collagenase activity of *P. gingivalis*. Leupeptin was used as a control inhibitor. A relative value of 100% was assigned to the degradation of type I collagen in the absence of the berry polyphenolic fraction. Results are expressed as the means ± SD of triplicate assays from three independent experiments. *Significant inhibition (*p* < 0.01) compared to the control.

**Figure 5:**
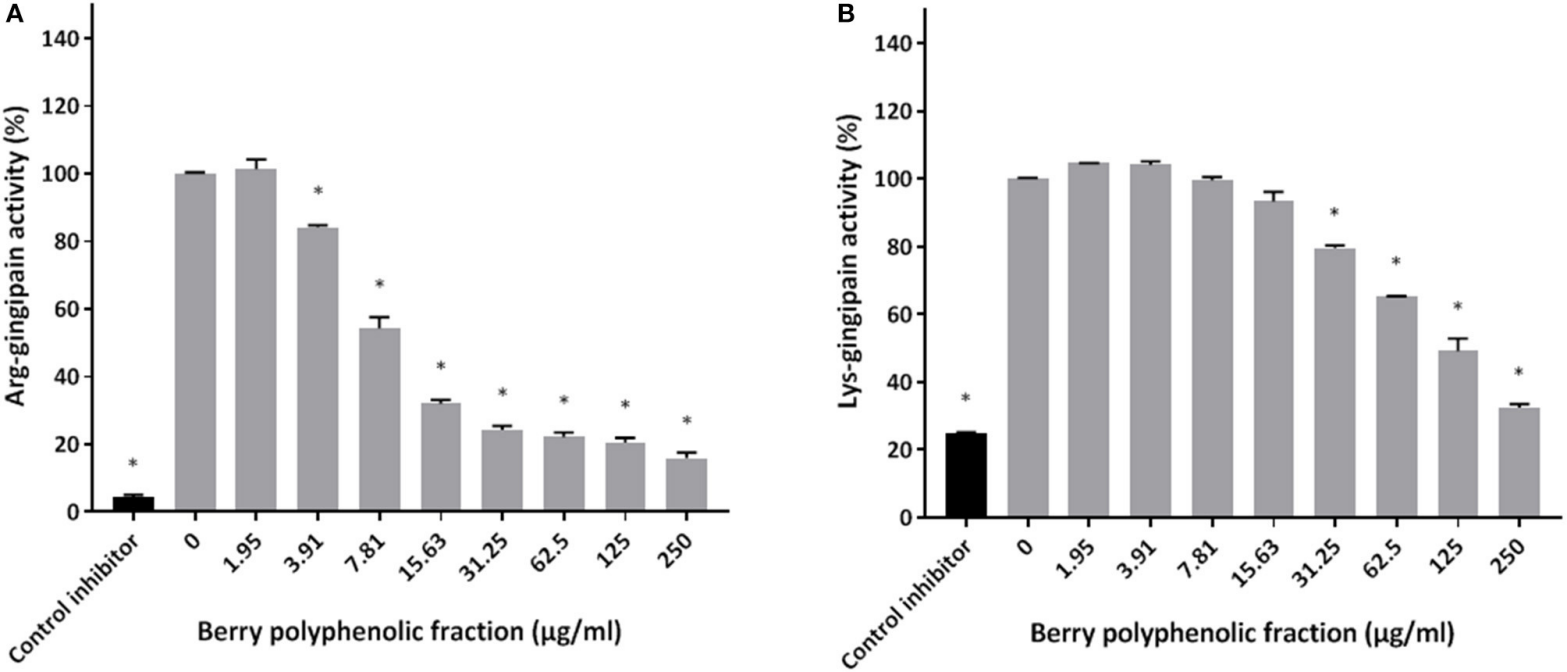
Effect of the berry polyphenolic fraction on the Arg-gingipain activity **(A)** and Lys-gingipain activity **(B)** of *P. gingivalis*. Tosyl-L-lysine chloromethyl ketone hydrochloride was used as a control inhibitor. A relative value of 100% was assigned to the activity in the absence of the berry polyphenolic fraction. Results are expressed as the means ± SD of triplicate assays from three independent experiments. *Significant inhibition (*p* < 0.01) compared to the control.

The effect of the berry polyphenolic fraction on type I collagen degradation by proteinases of *P. gingivalis* was evaluated. A significant dose-dependent inhibition occurred at concentrations ≥31.25 μg/ml, which decreased type I collagen degradation by 35.8% (Figure 4). We also tested the effect of the berry polyphenolic fraction on the activity of membrane-bound Arg- and Lys-gingipains of *P. gingivalis*. The fraction reduced in a dose-dependent manner the activity of Arg-gingipain (Figure 5A) and, to a lower degree, Lys-gingipain (Figure 5B). At the highest tested concentration (250 μg/ml), the fraction decreased the activity of Arg-gingipain by 84.2% and Lys-gingipain by 67.6%.

**Figure 6:**
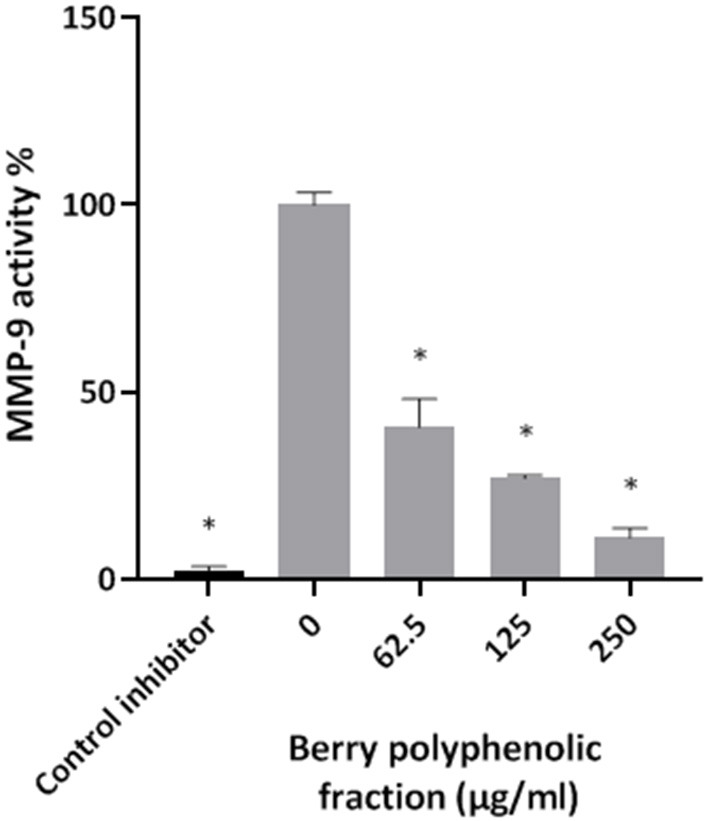
Effect of the berry polyphenolic fraction on MMP-9 activity. N-isobutyl-N-[4-methoxyphenylsulfonyl]glycyl hydroxamic acid was used as a control inhibitor. A relative value of 100% was assigned to the activity in the absence of the berry polyphenolic fraction. Results are expressed as the means ± SD of triplicate assays from three independent experiments. *Significant inhibition (*p* < 0.01) compared to the control.

Given that the berry polyphenolic fraction was found to be effective in inhibiting bacterial proteolytic enzymes, we hypothesized that it may also reduce the activity of host-derived MMPs that contribute to destruction of the periodontal tissues. The catalytic activity of MMP-9 used as a model was found to be dose-dependently inhibited by the berry polyphenolic fraction (Figure 6). At concentrations of 62.5, 125, and 250 μg/ml, the fraction decreased MMP-9 activity by 59.4, 72.9, and 88.8%, respectively.

**Figure 7:**
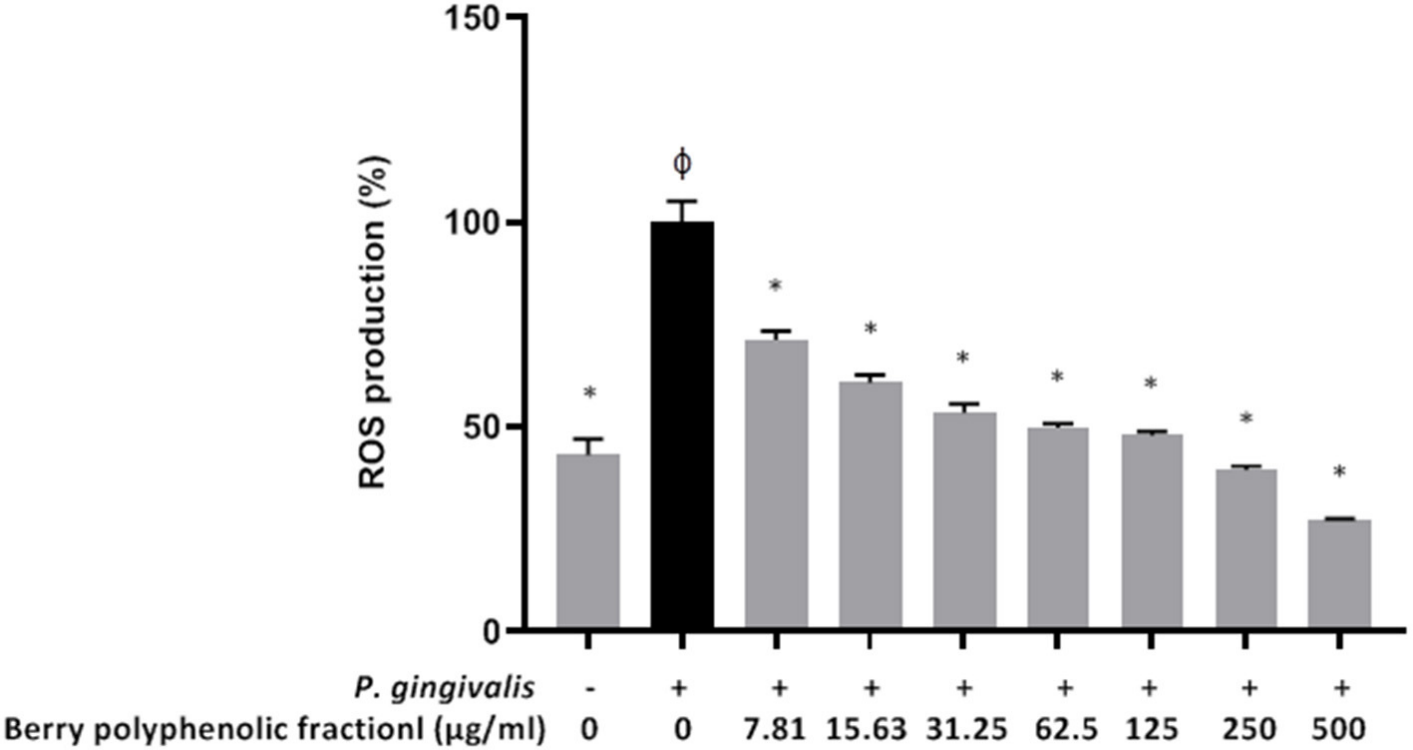
Effect of the berry polyphenolic fraction on *P. gingivalis*-induced ROS production by oral keratinocytes. The + symbol indicates the presence of *P. gingivalis* at MOI of 10^3^. A relative value of 100% was assigned to ROS production in the absence of the berry polyphenolic fraction. ^φ^Significant increase compared to the control (no *P. gingivalis* stimulation). Results are expressed as the means ± SD of triplicate assays from three independent experiments. ^φ^Significant increase compared to the control (no *P. gingivalis* stimulation). *Significant inhibition (*p* < 0.01) compared to the control (no fraction + *P. gingivalis* stimulation).

In a previous study [22], we showed that at concentrations up to 500 μg/ml, the berry polyphenolic fraction did not significantly decrease the oral keratinocyte (B11 cell line) viability in a colorimetric MTT test. Treating the oral keratinocytes with *P. gingivalis* (MOI of 10^3^) up-regulated ROS production by 2.3-fold (Figure 7). However, in the presence of the berry polyphenolic fraction (7.81–500 μg/ml), the *P. gingivalis*-mediated ROS production was significantly attenuated (Figure 7).

**Figure 8:**
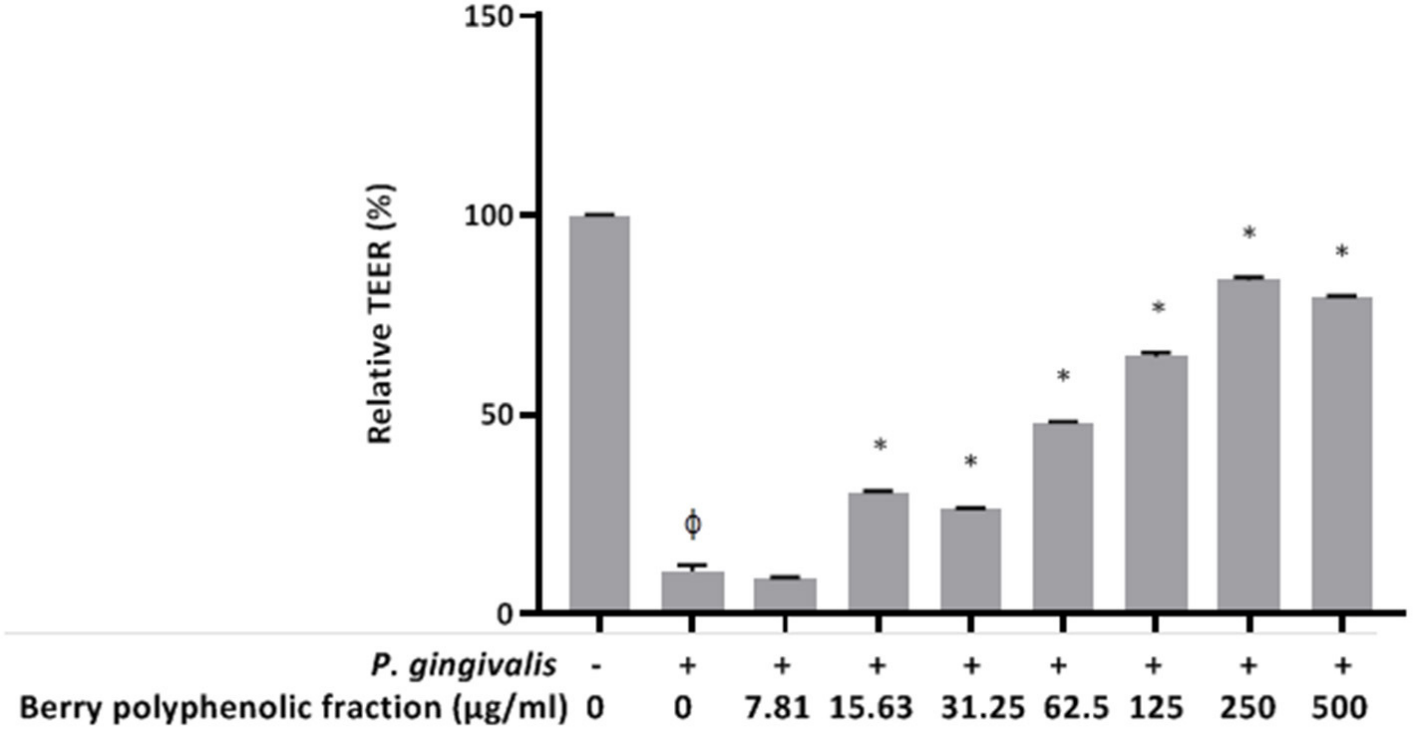
Effect of the berry polyphenolic fraction on *P. gingivalis*-mediated loss of oral keratinocyte barrier function. The + symbol indicates the presence of *P. gingivalis* at MOI of 10^3^. A relative value of 100% was assigned to the TEER recorded in the absence of *P. gingivalis* and the fraction. Results are expressed as the means ± SD of triplicate assays from three independent experiments. ^φ^Significant decrease (*p* < 0.001) compared to unstimulated control cells. *Significant increase (*p* < 0.01) compared to *P. gingivalis*-stimulated cells not treated with the fraction.

Since *P. gingivalis* has been shown to induce breakdown of keratinocyte barrier integrity (23, 25, 26), we investigated whether the berry polyphenolic fraction protects oral keratinocytes from the deleterious effect. A treatment (24 h) of keratinocytes with *P. gingivalis* (MOI of 10^3^) significantly reduced the TEER value by 89.25% (Figure 8). However, the berry polyphenolic fraction provided significant protection against the *P. gingivalis*-modulated reduction in TEER.

## Discussion

*P. gingivalis* produces a wide range of pathogenic determinants (adhesins, proteases, hemolysin, etc.) that contribute to periodontal tissue colonization and destruction as well as to immune defense evasion [6, 7]. Moreover, in infected periodontal pockets, *P. gingivalis* LPS up-regulates the expression of pro-inflammatory mediators and MMPs by host mucosal and immune cells thus promoting the continuous inflammation that modulates bone resorption [26]. Since *P. gingivalis* is a critical player in the pathogenesis of periodontitis, it represents a key bacterial target in view of effective preventive and therapeutic strategies directed against this disease.

Targeting virulence factors to disarm bacterial pathogens has been proposed as a promising anti-infective strategy [10, 11]. Such an approach offers advantages over traditional antibiotics by protecting the commensal microbiota from elimination and applying less selective pressure that leads to the development of resistance. In this respect, the aim of the present study was to investigate the ability of a berry fruit (cranberry, wild blueberry, strawberry) polyphenolic preparation, commercialized as Orophenol®, to attenuate the pathogenic properties of *P. gingivalis*. The fraction contains various classes of polyphenolic compounds, including phenolic acids, flavonols, flavan-3-ols, anthocyanins and procyanidins [22], which may act synergistically [27], likely bolstering the beneficial properties.

The berry polyphenolic fraction significantly reduced the growth *P. gingivalis*, although no complete inhibition was observed even at the highest tested concentration (2000 μg/ml). While the exact mechanism of antibacterial action of polyphenols is still unclear, evidence have been brought that most polyphenols interact with the bacterial surface, leading to disruption of membrane integrity and the release of essential intracellular constituents [28–30].

The capacity of *P. gingivalis* to lyse erythrocytes in infected and inflamed periodontal pockets allows the bacterium to acquire heme to survive and multiply *in vivo* [31]. Interestingly, the berry polyphenolic fraction completely inhibited the hemolytic activity of *P. gingivalis* and as such, may impede its ability to proliferate. Although the mechanism involved in the inhibition of the *P. gingivalis* hemolytic activity by the berry polyphenolic fraction has not been investigated, it may be related to structural changes in erythrocyte membranes caused by the polyphenols thus limiting the binding of the hemolysin, as previously reported in *Staphylococcus aureus* [32].

The adhesion of *P. gingivalis* to the oral mucosa by binding to extracellular matrix proteins is a prerequisite for the subsequent invasion of periodontal tissues [1, 7, 33]. We showed that the berry polyphenolic fraction decreases the adhesion of *P. gingivalis* to a basement membrane matrix model (Matrigel®), which may impair its ability to colonize subgingival areas. This anti-adherence property is in agreement with the results reported by Souissi et al. [22], who showed that the berry polyphenolic fraction inhibits the adherence of the cariogenic bacterium *Streptococcus mutans* to saliva-coated hydroxyapatite and nickel-chrome alloy.

*Porphyromonas gingivalis* proteinases may play central roles for allowing the bacterium to colonize gingival tissues, acquire essential nutrients, evade the host immune defenses, and destroy periodontal tissues [8, 9]. Given this, bioactive molecules with the capacity to inhibit *P. gingivalis* proteinases may represent promising anti-virulence agents. We investigated the ability of the berry polyphenolic fraction to reduce or inhibit the proteolytic activities of *P. gingivalis*. We showed that the fraction is effective in preventing the degradation of collagen by *P. gingivalis*. It also inhibited the membrane-bound Arg- and Lys-gingipains.

Given this anti-proteinase activity of the berry polyphenolic fraction and since MMPs are also actively involved in destruction of the connective tissue of the periodontium [34–36], we also assessed the effect of the fraction on MMP activity. Since MMP-9 activity increases in inflamed periodontitis sites and correlates with the severity of the disease [34–36], it was used as a model of MMP. We showed that the fraction significantly inhibits the catalytic activity of MMP-9.

During chronic periodontal inflammation, excessive ROS production by mucosal and immune cells may cause damages to DNA, proteins, and lipids, thus inducing cell death [37, 38]. We used an oral keratinocyte model to demonstrate that *P. gingivalis* induces the generation of intracellular ROS and that the berry polyphenolic fraction significantly inhibits ROS production by *P. gingivalis-*stimulated keratinocytes.

The oral epithelium is a physical barrier that protects the underlying gingival connective tissues against invasion by periodontal pathogens [39, 40]. Previous studies have shown that *P. gingivalis* can damage cell-to-cell junctions and consequently cause disruption of epithelial barrier function [25, 41]. This may contribute to the ability of periodontal pathogens to reach the connective tissues, enter the bloodstream, and induce several systemic disorders, including atherosclerosis, Alzheimer's disease, and rheumatoid arthritis [4, 5, 42]. We showed that the berry polyphenolic fraction can preserve the integrity and function of the oral keratinocyte barrier by limiting the decrease in TEER induced by a *P. gingivalis* challenge. Part of the protective effect is likely dependent on the ability of the fraction to inhibit the gingipains of *P. gingivalis*, which have been found to cleave epithelial junction transmembrane proteins, including occludin, E-cadherin, and β1-integrin [43].

In conclusion, the berry polyphenolic fraction (Orophenol®) investigated in the present study attenuated several pathogenic properties of *P. gingivalis*. Polyphenols in this wild blueberry, cranberry, and strawberry fraction may thus be promising bioactive molecules, which likely act in synergy, to prevent and/or treat periodontal disease. Future studies aimed at investigating the benefits yielded by using oral hygiene products or slow releasing devices (inserted in diseased periodontal pockets) containing the fraction need to be undertaken.

## Data Availability Statement

The original contributions presented in the study are included in the article/supplementary material, further inquiries can be directed to the corresponding author/s.

## Ethics Statement

Ethical review and approval was not required for this study in accordance with the local legislation and institutional requirements.

## Author Contributions

DG: conceptualization, formal analysis, funding acquisition, project administration, supervision, and writing. ABL and KV: investigation and methodology. All authors contributed to the article and approved the submitted version.

## Funding

This study was financially supported by the Laboratoire de Contrôle Microbiologique of Université Laval.

## Conflict of Interest

The authors declare that the research was conducted in the absence of any commercial or financial relationships that could be construed as a potential conflict of interest.

## Publisher's Note

All claims expressed in this article are solely those of the authors and do not necessarily represent those of their affiliated organizations, or those of the publisher, the editors and the reviewers. Any product that may be evaluated in this article, or claim that may be made by its manufacturer, is not guaranteed or endorsed by the publisher.
